# RETRACTION: Short-Chain Fatty Acid Propionate Alleviates Akt2 Knockout-Induced Myocardial Contractile Dysfunction

**DOI:** 10.1155/jdr/9829275

**Published:** 2025-12-01

RETRACTION: L. Li, Y. Hua, J. Ren, “Short-Chain Fatty Acid Propionate Alleviates Akt2 Knockout-Induced Myocardial Contractile Dysfunction,” *Journal of Diabetes Research*, 2011, 851717, https://doi.org/10.1155/2012/851717

The above article, published online on 22 September 2011 in Wiley Online Library (http://wileyonlinelibrary.com), has been retracted by agreement between the journal Section Editor, Bright Starling Emerald, and John Wiley & Sons Ltd. The retraction has been agreed due to multiple concerns with Figures 3, 4 and 6 being initially raised by a third party. On further investigation, including concerns raised on PubPeer [1], further figure concerns were noted:

• Similarity between the WT panel in Figure 3a, and the MyAKT panel in Figure 9a of [2]
• Similarity between the WT-propionate panel in Figure 3a, and the TUDCA panel in Figure 9a of [2]
• Similarity between the AKTKO-propionate panel in Figure 3a, and the WT panel in Figure 9a of [2]
• Similarity between the AKTKO panel in Figure 3a, and the FVB-STZ panel in Figure 7 k of [3]
• The pPTEN bands shown in Figure 4d appear to be similar to the B-Actin bands shown in Figure 6b, when rotated 180 degrees.
• The pPTEN bands shown in Figure 4d appear to be similar to the B-Actin bands shown in Figure 6c, when rotated 180 degrees.
• The B-Actin bands shown in Figures 6b, 6c and 6d appear to be identical.

The authors were unresponsive when asked for an explanation, and after assessment by the Editorial Board the article is retracted.
